# Construction and validation of a predictive model for the risk of bowel resection in adults with incarcerated groin hernia

**DOI:** 10.1186/s12893-023-02245-7

**Published:** 2023-12-11

**Authors:** Zheqi Zhou, Yujie Li, Bin Li, Likun Yan, Yingying Lei, Cong Tong

**Affiliations:** 1 Department of General Surgery, Shaanxi Provincial People’s Hospital, Xi’an, 710068 China; 2 Yan’an University, Yan’an, 716000 China; 3 Tianjin Medical University, Tianjin, 300052 China; 4 Department of Gynecology, Shaanxi Provincial People’s Hospital, Xi’an, 710068 China

**Keywords:** Inguinal hernia, Risk factors, Bowel resection, Constriction of the bowel, Ischemic necrosis

## Abstract

**Background:**

It is difficult to definitively determine the degree of ischemia in the bowel in which an incarcerated groin hernia is embedded. Failure to diagnose and intervene promptly and accurately increases the rate of bowel resection and patient mortality. The aim of this study is to investigate the risk factors for incarcerated inguinal hernia complicating bowel necrosis with resection and to establish a predictive model as a reference for clinical work.

**Methods:**

Patients with incarcerated groin hernia who were admitted to our hospital were retrospectively analyzed. They were divided into bowel resection and non-bowel resection groups based on whether bowel resection was performed in the surgical record and postoperative pathological results. Risk factors for the development of bowel resection in incarcerated groin hernia were analyzed by univariate analysis and multivariate logistic regression, respectively. The screened independent risk factors were used to establish a prediction model, and finally, the predictive ability and accuracy of the model were validated and the clinical benefit was analyzed.

**Results:**

A total of 345 patients with incarcerated groin hernia were included, of whom 58 underwent bowel resection for bowel necrosis and 287 did not. Multifactorial logistic regression analysis identified bowel obstruction (OR, 7.285 [95% CI, 2.254–23.542], P = 0.001), peritonitis (OR, 16.786 [95% CI, 5.436–51.838], P = 0.000), duration of incarcerated groin hernia (OR, 1.009 [95% CI, 1. 001-1.018], P = 0.034), heart rate (OR, 1.109 [95% CI, 1.021–1.205], P = 0.014), and preoperative total protein (OR, 0.900 [95% CI, 0.836–0.969], P = 0.005) were independent risk factors for bowel resection in incarcerated groin hernia. The predictive value of the established prediction model was basically in agreement with the measured value with a consistency index of 0.938 (0.901–0.974) and had a good clinical benefit.

**Conclusion:**

Clinical screening and management of independent risk factors for bowel resection in patients with incarcerated groin hernia should be strengthened. The predictive model developed in this study has high diagnostic efficacy for bowel resection associated with incarcerated inguinal hernia, with the aim of reducing the incidence of bowel resection and unplanned secondary surgery.

## Introduction

Severe incarcerated groin hernia (IGH) can lead to bowel obstruction or even bowel strangulation followed by bowel necrosis, seriously affecting the life and health of patients. According to statistics, about 15% of IGH patients eventually develop bowel necrosis and undergo bowel resection, with a mortality rate of about 5% [1, 2]. Emergency surgical treatment is one of the effective methods to avoid bowel necrosis [3, 4], but to date, it is still difficult to clearly determine the degree of ischemia of the embedded bowel in clinical work [5], and the inability to accurately and timely diagnose and intervene will increase the rate of bowel resection, the rate of complications, and the mortality rate of patients [6]. Previous studies on the analysis of risk factors for IGH-induced bowel resection have limitations in the variables included and lack the construction of preoperative clinical prediction models. This study will investigate the independent risk factors for bowel resection in IGH and develop a predictive model. This study will provide clinicians with a predictive model for the development of bowel resection in patients with IGH to assist the operator in selecting the optimal surgical and therapeutic modality, and to reduce the rate of bowel resection and the risk of unplanned secondary surgery in conjunction with intraoperative judgments of bowel viability.

## Materials and methods

### Study design and patient selection

A retrospective analysis of 345 IGH patients who visited Shaanxi Provincial People’s Hospital from January 2010 to January 2023 was performed. Patients were divided into a training set and a validation set according to the time of visit and inpatient department. Among them, 257 patients whose inpatient department was general surgery from 2016 to 2023 were used as the training set, and the model was trained. The 88 patients whose inpatient department was emergency surgery from 2010 to 2015 were used as validation to test the model. Cases included in the study were categorized into bowel resection (BR) and non-bowel resection (NBR) groups based on whether or not bowel resection was performed in patients with IGH in the surgical records and postoperative pathologic findings. The NBR group was defined as those with a preoperative diagnosis of IGH in whom intraoperative exploration revealed normal bowel perfusion or in whom perfusion was restored after a period of observation without the need for bowel resection; and the BR group was defined as those with a preoperative diagnosis of IGH in whom intraoperative exploration revealed ischemic tissue necrosis and bowel resection, and postoperative pathology confirmed bowel necrosis. This study was conducted in accordance with the tenets of the Declaration of Helsinki and current ethical guidelines and was approved by the Ethics Committee of XXX Hospital. Informed consent was obtained from patients and their families. Inclusion criteria were as follows: (1) age 18–100 years; (2) complete clinical information; and (3) admitted with a diagnosis of IGH. Exclusion criteria were as follows: (1) those who did not undergo surgery due to successful preoperative manipulation for reduction of the hernia sac; (2) those who did not undergo surgery or died due to other diseases; (3) intraoperative bowel resection for non-incarcerated factors; (4) those whose incarcerated tissue was not the bowel; and (5) those who underwent unplanned secondary surgery due to misjudgment of the activity of the incarcerated bowel.

### Observed indicators

The clinical data of all included patients with IGH were retrieved from the hospital information system (HIS) and retrospectively analyzed. Specific information included sex, age, length of hospital stay, underlying disease, history of incarceration, history of abdominal surgery, history of smoking, history of alcohol abuse, length of incarceration, number of deliveries in female patients, presence of bowel obstruction (diagnosed by imageologic examination), preoperative peritonitis, preoperative imageologic examination (CT or B-ultrasound for exploration of hernia), peritonitis, preoperative imaging (CT or B-ultrasound to assess hernia sac volume and internal ring size), preoperative laboratory tests such as white blood cell count, neutrophil count, lymphocyte count, neutrophil-lymphocyte ratio (NLR), and preoperative total protein.

### Statistical analysis

SPSS 18.0 software was used for statistical analysis. Continuous variables are expressed as mean ± standard deviation or median (interquartile range, IQR) and compared by Student’s t test (for normally distributed continuous variables) or independent Mann-Whitney U test (for non-normally distributed continuous variables), and categorical variables were compared by χ2 test. A p-value < 0.05 was considered statistically significant. Variables with *P* < 0.05 were screened out and included in multivariate logistic regression for a second screening, and the screened independent risk factors were next used as final variables in the construction of a risk prediction model to predict the risk of bowel resection in patients with IGH, and a nomogram was created using R (R 4.2.1) (http://www.R-project.org), using the rms program package to build a nomogram prediction model. Internal validation of 10-fold cross-validation and bootstrap validation methods were performed using the caret package, and the consistency index (C-index) was calculated using the rms package. The ROCR and rms packages were used to construct the receiver operating characteristic (ROC) curve. Decision curve analysis (DCA) was used to further evaluate the predictive performance. The difference was considered statistically significant at *P* < 0.05.

## Results

Results of univariate analysis of clinical data in the 2 groups.

As shown in Table 1, data were collected from 345 patients enrolled for IGH, including 257 patients in the training set and 88 patients in the validation set. Patients in the training and validation sets had statistically significant differences in history of incarceration, heart disease, chronic obstructive pulmonary disease (COPD), length of stay, internal ring mouth diameter, hernia sac volume/hernia ring internal diameter, neutrophil leukocyte count, and lymphocyte count. In the training set, 41 cases had bowel necrosis due to IGH and 216 cases did not have bowel necrosis. There were statistically significant differences between the two groups in the training set in terms of sex, bowel obstruction, obvious peritonitis, age, heart rate, duration of IGH, length of stay, hernia sac volume/hernia ring internal diameter, white blood cell count, neutrophil count, lymphocyte count, neutrophil to lymphocyte ratio (NRL), preoperative total protein, and number of deliveries in female patients.

**Table 1:** Results of single factor analysis

Variables	Total (n = 345)				Training cohort (n = 257)		
	Training set (n = 257)	Validation set (n = 88)	*P*-value*		No bowel resection (n = 216)	Bowel resection (n = 41)	*P*-value*
**Sex**			0.099				0.004*
Male	191(74.32%)	73(82.95%)			168(77.78%)	23(56.10%)	
Female	66(25.68%)	15(17.05%)			48(22.22%)	18(43.90%)	
**History of incarceration**			0.041*				0.516
Without	242(94.16%)	77(87.50%)			202(93.52%)	40(97.56%)	
With	15(5.84%)	11(12.50%)			14(6.48%)	1(2.44%)	
**Recurrent hernia**			0.109				0.804
Nonrecurrent	239(93.00%)	77(87.50%)			200(92.59%)	39(95.12%)	
Recurrent	18(7.00%)	11(12.50%)			16(7.41%)	2(4.88%)	
**Bowel obstruction**			0.731				<0.001*
Without	179(69.65%)	63(71.59%)			170(78.70%)	9(21.95%)	
With	78(30.35%)	25(28.41%)			46(21.30%)	32(78.05%)	
**Peritonitis**			0.997				<0.001*
Without	222(86.38%)	76(86.36%)			205(94.91%)	17(41.46%)	
With	35(13.62%)	12(86.36%)			11(5.09%)	24(58.54%)	
**Hernia type(Insertion side)**			0.073				0.433
Indirect	182(70.82%)	74(84.09%)			153(70.83%)	29(70.73%)	
Direct	44(17.12%)	9(10.23%)			39(18.06%)	5(12.20%)	
Femoral	31(12.06%)	6(6.82%)			24(11.11%)	7(17.07%)	
**Smoking history**			0.632				0.099
Without	186(72.37%)	66(75.00%)			152(70.37%)	34(82.93%)	
With	71(27.63%)	22(25.00%)			64(29.63%)	7(17.07%)	
**History of alcohol consumption**			0.654				0.271
Without	210(81.71%)	70(79.55%)			174(80.56%)	36(87.80%)	
With	47(18.29%)	18(20.45%)			42(19.44%)	5(12.20%)	
**History of increased intra-abdominal pressure disease**			0.105				0.772
Without	223(86.77%)	82(93.18%)			188(87.04%)	35(85.37%)	
With	34(13.23%)	6(6.82%)			28(12.96%)	6(14.63%)	
**Hypertension**			0.677				0.064
Without	187(72.76%)	62(70.45%)			162(75.00%)	25(60.98%)	
With	70(27.24%)	26(29.55%)			54(25.00%)	16(39.02%)	
**Diabetes**			0.851				0.516
Without	242(94.16%)	84(95.45%)			202(93.52%)	40(97.56%)	
With	15(5.84%)	4(4.55%)			14(6.48%)	1(2.44%)	
**hyperlipidemia**			0.503				1.000
Without	245(95.33%)	86(97.73%)			206(95.37%)	39(95.12%)	
With	12(4.67%)	2(2.27%)			10(4.63%)	2(4.88%)	
**Cerebral infarction**			0.914				0.772
Without	226(87.94%)	77(87.50%)			191(88.43%)	35(85.37%)	
With	31(12.06%)	11(12.50%)			25(11.57%)	6(14.63%)	
**Heart Disease**			<0.001*				0.250
Without	188(73.15%)	85(96.59%)			161(74.54%)	27(65.85%)	
With	69(26.85%)	3(3.41%)			55(25.46%)	14(34.15%)	
**Cancer**			1.000				1.000
Without	253(98.44%)	86(97.73%)			213(98.61%)	40(97.56%)	
With	4(1.56%)	2(2.27%)			3(1.39%)	1(2.44%)	
**COPD**			0.023*				0.225
Without	227(88.33%)	85(96.59%)			188(87.04%)	39(95.12%)	
With	30(11.67%)	3(3.41%)			28(12.96%)	2(4.88%)	
**Age (years), mean ± SD**	65.00(54.00–78.00)	70.00 (51.00–78.00)	0.652		63.00(53.00–77.00)	77.00(64.00–80.00)	0.002*
**Heart rate (beats/min), mean ± SD**	78.00(74.00–80.00)	80.00(72.50-83.75)	0.282		78.00(74.00–80.00)	78.00(70.50–78.00)	0.009*
**Duration of hernia (years), mean ± SD**	2.00(0.16-10.00)	3.00 (0.20-8.00)	0.526		2.00(0.16–9.75)	2.00(0.07-10.00)	0.632
**Duration of incarcerated hernia(hour), mean ± SD**	12.00(3.00–24.00)	10.00(5.00–24.00)	0.840		7.50(3.00–24.00)	48.00(18.00–72.00)	<0.001*
**Length of stay (days), mean ± SD**	7.00(4.00–10.00)	9.00(6.00–12.00)	0.002*		6.00(4.00–9.00)	14.00(10.50–17.50)	<0.001*
**Number of deliveries in female patients, mean ± SD**	2.00(1.75-3.00)	2.00(1.00–4.00)	0.417		2.00(1.00–2.00)	3.00(2.00–5.00)	0.003*
**Hernia sac volume(cm** ^**3**^ **), mean ± SD**	60.00(29.19–192.00)	90.00(48.00-198.00)	0.117		40.76(22.11-112.47)	101.52(31.82–432.00)	0.092
**Inner ring mouth inner diameter, mean ± SD**	1.50(1.00–2.00)	1.20(0.80–1.78)	0.003*		1.50(1.00-2.18)	1.20(0.83–1.85)	0.068
**Hernia sac volume/hernia ring inner diameter, mean ± SD**	45.00(22.99-134.06)	92.33(33.18-180.47)	0.006*		40.76(22.11-112.47)	93.53(30.24-221.77)	0.015*
**White blood cell count (×10** ^**9**^ **/L), mean ± SD**	7.44(5.63–9.68)	7.80(6.11–10.96)	0.232		7.28(5.53–9.37)	9.13(7.10-13.08)	<0.001*
**Neutrophilic leukocyte count (×10** ^**9**^ **/L), mean ± SD**	5.40 (3.78–8.04)	1.14(0.83–1.68)	<0.001*		5.13(3.61–7.75)	7.63(5.31–11.17)	<0.001*
**Lymphocyte count (×10** ^**9**^ **/L), mean ± SD**	1.13(0.71–1.58)	5.95(4.08–8.70)	<0.001*		1.20(0.77–1.68)	0.80(0.53–1.12)	<0.001*
**Neutrophil to lymphocyte ratio(NLR), mean ± SD**	5.31(2.75–9.67)	5.83(2.86–9.38)	0.690		4.53(2.47–8.53)	9.54(6.08–15.22)	<0.001*
**Preoperative total protein, mean ± SD**	64.02 ± 8.85	63.96 ± 9.34	0.950		65.43 ± 8.11	56.59 ± 8.95	<0.001*

COPD Chronic Obstructive Pulmonary Disease, ∗ Statistically significant difference

### Results of multifactor logistic regression analysis

Multivariate logistic regression analysis was performed with the occurrence of bowel necrosis as the dependent variable and the *p* < 0.05 item in the univariate analysis of BR and NBR as the independent variable. The results are shown in Table 2 for the presence of bowel obstruction (OR, 7.285 [95% CI, 2.254–23.542], P = 0.001), peritonitis (OR, 16.786 [95% CI, 5.436–51.838], P = 0.000), duration of incarcerated groin hernia (OR, 1.009 [95% CI, 1. 001-1.018], P = 0.034), heart rate (OR, 1.109 [95% CI, 1.021–1.205], P = 0.014), and preoperative total protein (OR, 0.900 [95% CI, 0.836–0.969], P = 0.005) were independent risk factors for bowel resection in IGH.

**Table 2:** Binary logistic regression results

Variable	OR	95% CI	*P*-value*
Sex	0.914	0.298–2.802	0.875
Bowel obstruction	7.285	2.254–23.542	0.001*
Peritonitis	16.786	5.436–51.838	<0.001*
Age	1.015	0.974–1.058	0.472
Duration of incarcerated hernia	1.009	1.001–1.018	0.034*
Heart rate	1.109	1.021–1.205	0.014*
White blood cell count	1.166	0.726–1.872	0.524
Neutrophilic leukocyte count	1.132	0.656–1.952	0.656
Lymphocyte count	0.238	0.027–2.214	0.199
Neutrophil to lymphocyte ratio	0.938	0.792–1.112	0.462
Preoperative total protein	0.900	0.836–0.969	0.005*
Hernia sac volume/hernia ring inner diameter	1.000	0.999–1.002	0.657

95%CI = 95% confidence interval, OR = Odds ratio, ∗ statistically significant difference

### Predictive model building and validation

**Fig. 1:**
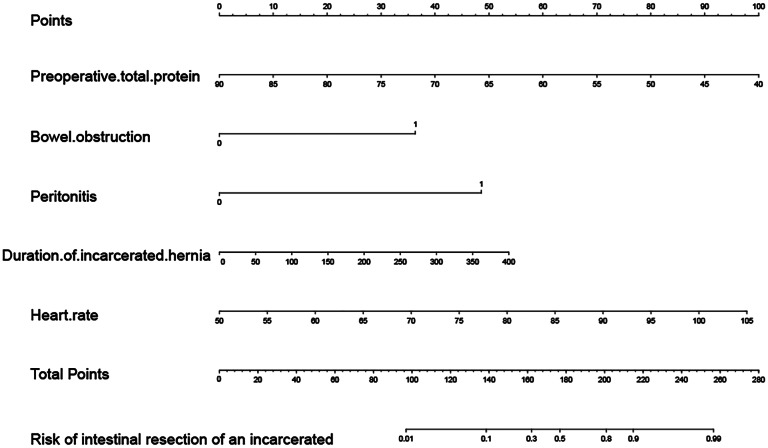
The nomogram for bowel resection in patients with IGH

**Fig. 2:**
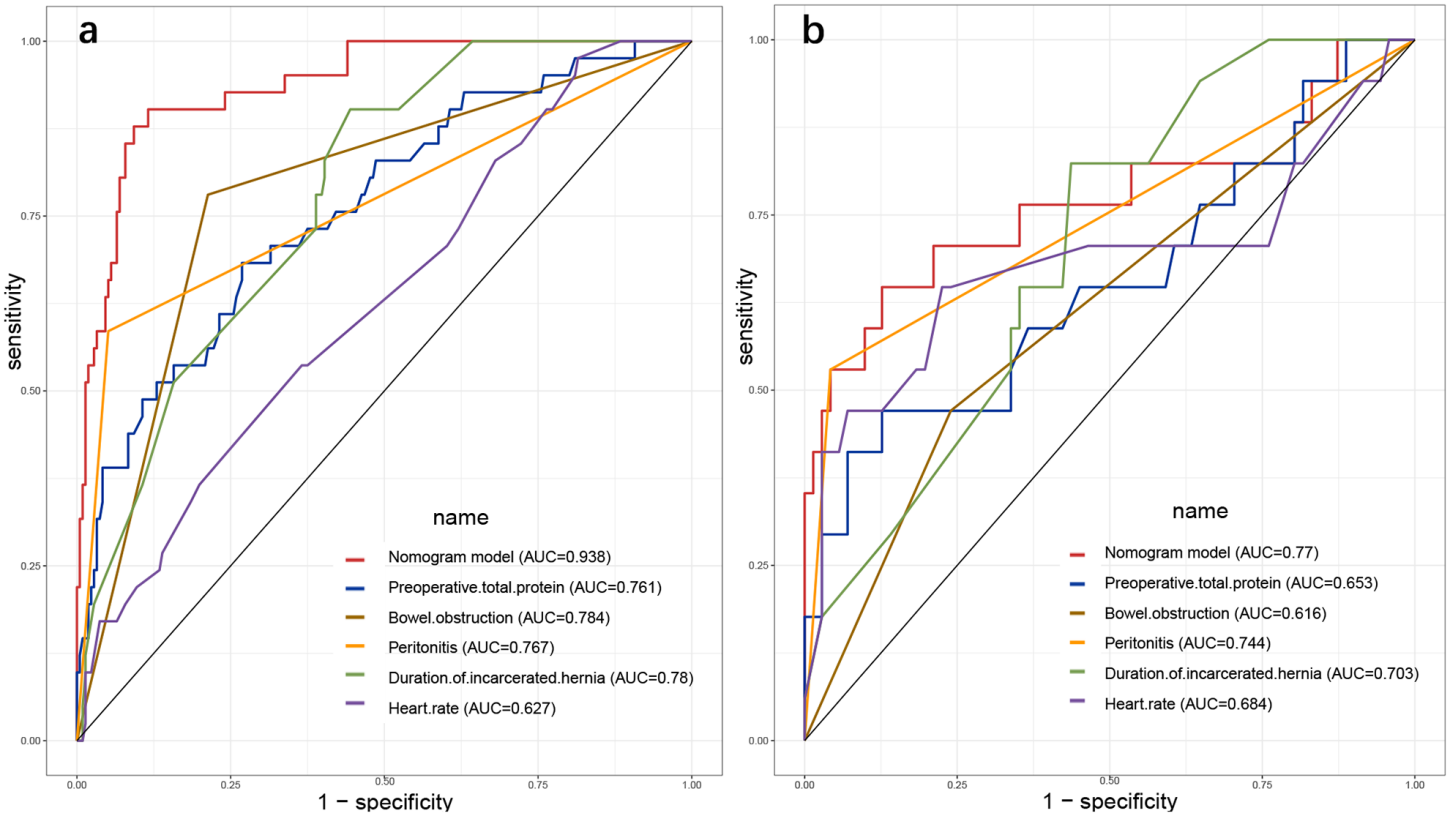
The AUROC for each variable and model. **a** training set; **b** validation set

**Fig. 3:**
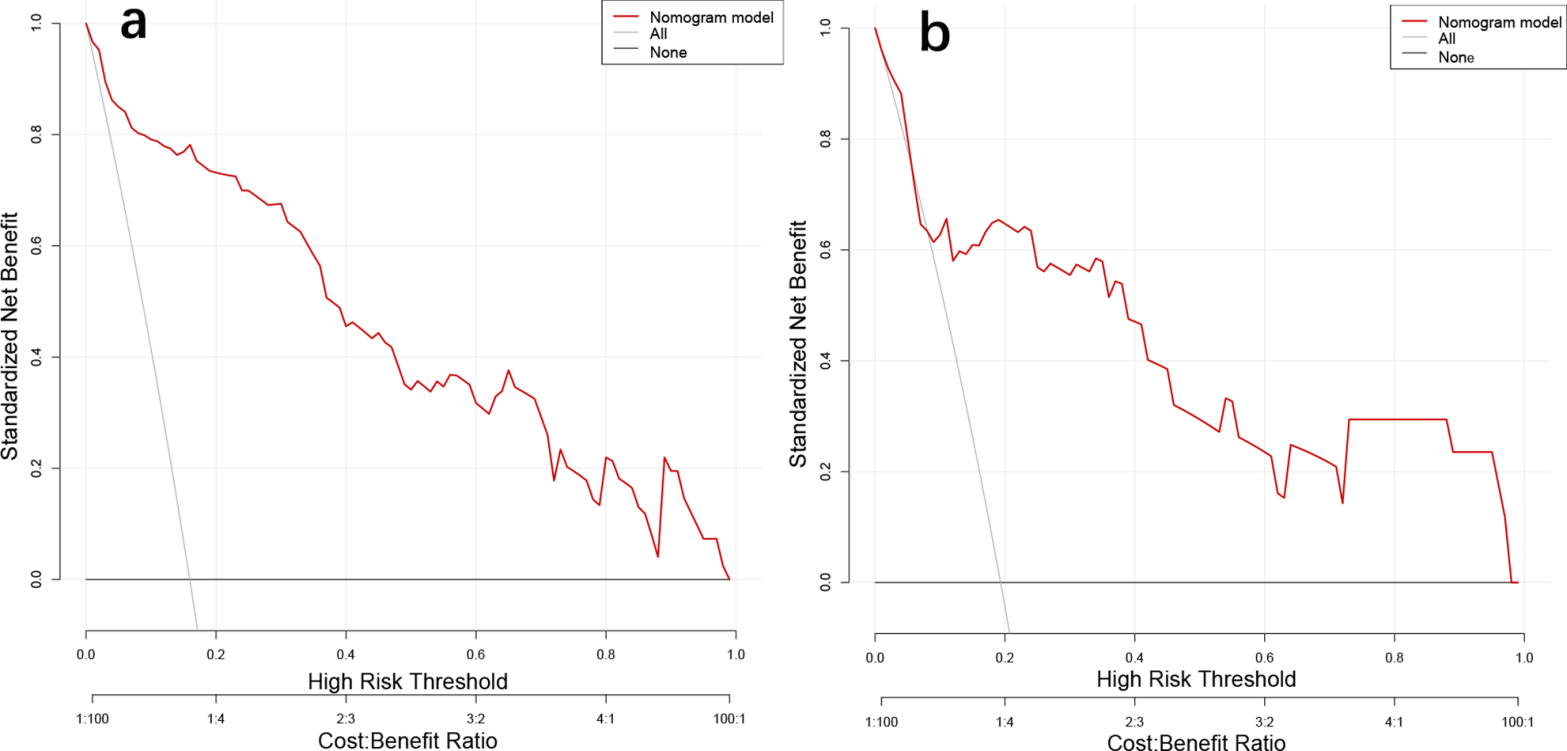
Decision curve analysis for predictive modeling

In this study, we developed a risk prediction model based on five independent risk factors for bowel resection for IGH and plotted the nomogram (Fig. 1). The C-index of this model was 0.938 (0.901–0.974). The area under the ROC curve (AUROC) was 0.938 (0.901–0.975) for the training set and 0.770 (0.612–0.927) for the validation set. The AUROC for each variable in the model and training set were, in descending order: nomogram (AUC = 0.938, *P *< 0.001), presence of bowel obstruction (AUC = 0.784, *P* < 0. 001), duration of IGH (AUC = 0.780, *P* < 0.001), and peritonitis (AUC = 0.767, *P* < 0.001), heart rate (AUC = 0.627, P = 0.010), and preoperative total protein (AUC = 0.239, *P* < 0.001) (Fig. 2). DCA in both the training and validation sets had large net gains at each threshold probability (Fig. 3). As shown in Table 3, the 10-fold cross-validation and bootstrap validation AUROC were 0.932 (0.924–0.940) and 0.935 (0.935–0.941), respectively.

Nomogram of the risk of bowel resection for IGH. Each variable is assigned a point on the top axis by drawing a line upward. The sum of these numbers is located on the total points axis, and a line is drawn downward to determine the risk of bowel necrosis requiring bowel resection in patients with IGH. In the figure, 1 means yes and 0 means no.

Model calibration curves for the training set and validation set. a Training set; b Validation set. The nomogram-predicted probability of risk of bowel resection for IGH is plotted on the x-axis, while the actual probability is plotted on the y-axis.

Decision curve analysis (DCA) of bowel resection for IGH. DCA depicting the net clinical benefit. a training set; b validation set. Black line = net benefit when no patient would experience the event; gray line = net benefit when all patients would experience the event.

**Table 3:** Internal validation results

Internal verification method	AUROC	95% CI
10-fold cross-validation	0.932	0.924–0.940
Bootstrap validation	0.935	0.935–0.941

AUROC Area Under the ROC Curve; 95% CI 95%CI = 95% confidence interval

## Discussion

In the results of univariate analysis of this study, the risk of bowel resection was higher in female patients with IGH than in males. The same results were found in some previous studies with univariate analysis results [7, 8]. In this study, femoral hernia was higher in female patients with IGH (37.04%), and femoral hernia was 36% in the group of female patients with bowel resection. The author believes that one of the reasons for this is the higher incidence of femoral hernias in female patients with inguinal hernias [9, 10] and the much higher risk of developing a narrow bowel in femoral hernias than in other types of hernias [11, 12]. However, the results of this study did not find gender to be an independent risk factor for bowel resection in patients with IGH. Bowel obstruction leads to increased pressure in the bowel lumen of the obstructed segment, which in turn increases blood flow disturbances in the bowel wall and induces bowel necrosis [13]. The present study found that the presence of preoperative symptoms of bowel obstruction increased the risk of bowel resection in patients with IGH, and the same results were obtained in a previous study [14]. Significant signs of peritonitis are the hallmark signs of bowel necrosis in patients with IGH, and the results of the present study reaffirm this finding [15]. Prolonged IGH increases the duration of bowel ischemia, resulting in the deposition of local tissue metabolites. This also promotes edema of the incarcerated bowel, which in turn increases compression of the bowel wall vessels by the inner ring, aggravating ischemia and hypoxia of the bowel canal and ultimately leading to bowel necrosis [16, 17]. The present study confirmed that the duration of IGH was an independent risk factor for bowel necrosis. This result is consistent with the findings of Ge BJ, Chen, P, Xie X, et al. [5, 8, 18]. The present study also found that low preoperative total protein was also an independent risk factor for bowel necrosis, because tissues are more susceptible to edema under low plasma osmolality, and in patients with IGH, the ischemic and hypoxic state of the incarcerated bowel aggravates the edema of the bowel wall, further aggravating the degree of ischemia. The current relevant study did not include preoperative total protein in the analysis, which was complemented by the results of the present study. Local inflammation induced by bowel strangulation and necrosis may cause inflammatory factors to stimulate sympathetic nerves and increase heart rate [19], even leading to severe systemic inflammatory response syndrome and endangering the patient’s life, all of which are independent risk factors for bowel strangulation in IGH.

Most studies on women’s deliveries and hernias have mainly focused on pregnancy-related IGH formation and recurrence. However, there is a lack of research on IGH and bowel resection caused by IGH [20]. In this study, we statistically analyzed the number of deliveries in female patients with IGH and found that the number of deliveries in female patients with IGH was higher in BR than in NBR, and the difference was statistically significant. We believe this finding suggests that the number of deliveries in female patients may be a potential risk for bowel resection due to IGH, which may be due to the larger diameter of the hernia ring in female patients with a high number of deliveries, resulting in easier access to the bowel within the hernia sac, and the immediate edema of the intestinal wall caused by minor incarcerations, which may lead to incarceration. However, due to the small number of female patients, this study should be further investigated in a multicenter follow-up study with a large sample. In this study, we counted the hernia sac volume and the size of the internal ring in two groups of patients. There is no accepted method for calculating the hernia sac volume, and we roughly expressed it by the results of length*width*height, but we found only a significant difference in the ratio of the inner ring and the hernia sac volume to the inner ring, while in the results of multivariate logistic regression analysis, the ratio of the inner ring and the hernia sac volume to the inner ring was not an independent risk factor. Patient age was also not an independent risk factor for bowel resection of IGH in our results. However, there is considerable controversy about this issue. Previous studies have found that advanced age is a risk factor for bowel resection in IGH [12, 18], possibly because advanced IGH patients with bowel resection present early with atypical signs and symptoms, leading to delayed treatment and surgery. It has also been shown that advanced age is not a risk factor for bowel resection in patients with IGH [5, 8]. NLR is an independent risk factor for bowel resection in some studies [5, 8, 18], but in this study a statistically significant difference was found only in univariate analysis, which may be due to the small sample size included. To date, it remains inconclusive whether the degree of increase in inflammatory markers may indicate a high risk of bowel resection [8]. In addition, our findings confirm that bowel resection in IGH increases the length of hospital stay of patients.

The surgical approach for IGH has been debated, with some scholars arguing that open surgery should be performed in patients with severe symptoms and a high likelihood of necrosis of the incarcerated tissue, and others arguing that laparoscopic surgery is the preferred surgical approach for IGH [21, 22]. In surgery for IGH, blood flow to the incarcerated bowel is an important factor in intraoperative decision making, and irreversible ischemia can lead to bowel necrosis [23]. The advantages of laparoscopy over open surgery are that it is easier to observe the recovery of bowel color and peristalsis after retraction to confirm bowel viability, and it facilitates secondary evaluation of the retrieved bowel at the end of surgery to avoid unnecessary organ removal [24]. In the surgical management of IGH complicated by bowel necrosis, the goal is to restore and monitor the activity of the hernia contents, remove necrotic tissue, and reduce the risk of patient death [25]. The author believes that emergency surgery is necessary for IGH that cannot be returned by manipulation, but it is very difficult for surgeons to determine whether bowel resection is due to intraoperative misjudgment of bowel activity, which may increase the rate of bowel resection or unplanned secondary surgery. In the surgical treatment of bowel necrosis in IGH, the patient’s condition, the surgeon’s surgical technique, and the surgical conditions of the attending medical institution should be fully integrated to reasonably select the appropriate surgical modalities, such as traditional hernia repair, tension-free hernia repair, and translaparoscopic hernia repair, as well as the correct treatment of the patient’s incarcerated bowel to avoid bowel resection and unplanned secondary surgery as much as possible.

**Fig. 4:**
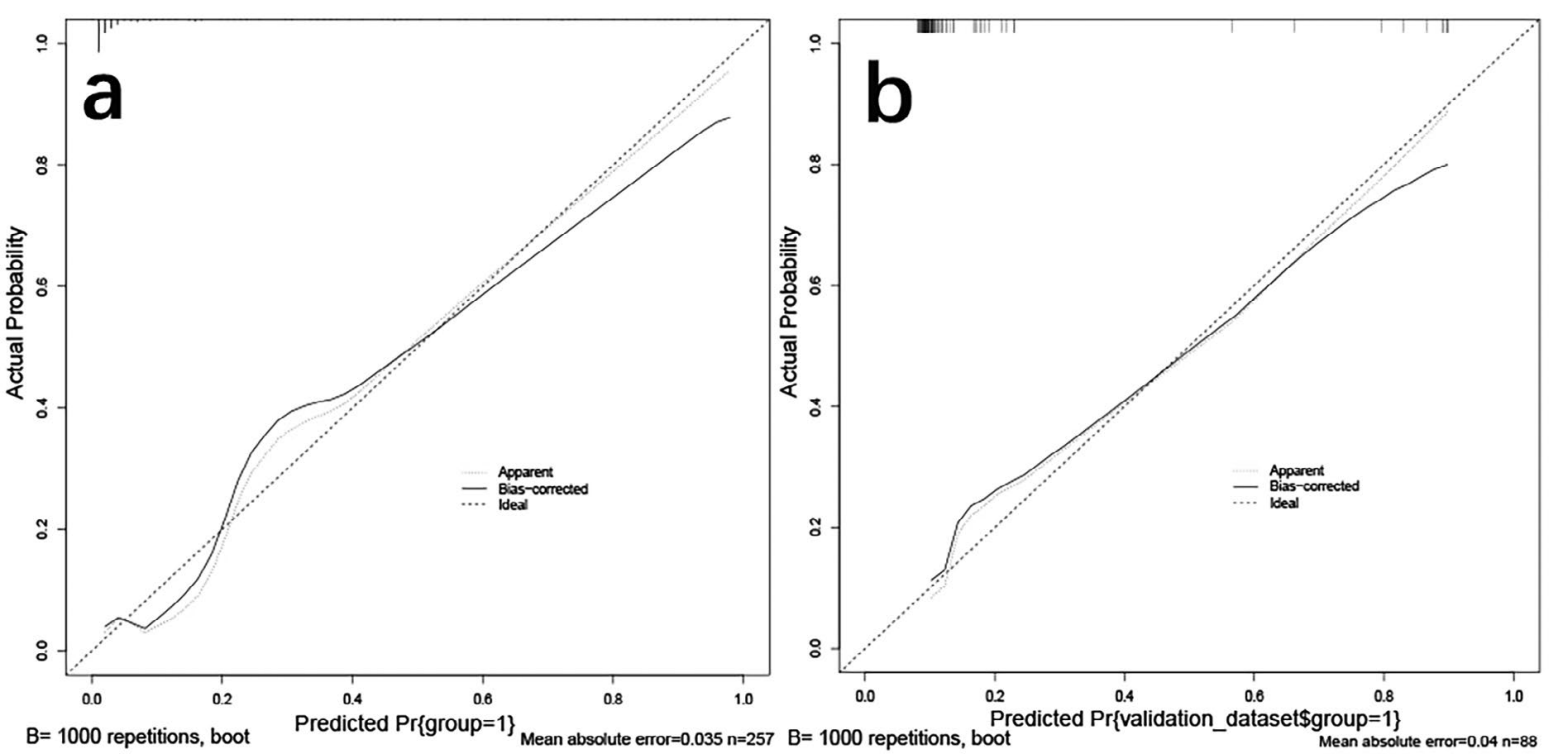
Calibration curves for predictive models

The prediction model developed in this study has a high C-index of 0.938 (0.901–0.974). The calibration curves of the training and validation sets show that there is a good agreement between the predictions of the model and the actual observations (Fig. 4). The model has the largest AUROC compared to the other variables in the validation and training sets. The DCA of the training and validation sets showed that the application of the model to predict the risk of IGH bowel necrosis was highly rewarding over a wide range of threshold probabilities (Fig. 3). In addition, both 10-fold cross-validation and bootstrap validation yielded good AUROC, confirming the good predictive performance and generalization ability of the model. These results suggest that the prediction model developed in this study has good predictive performance and is an ideal model for predicting bowel resection in IGH patients.

The main limitations of our study are (1) the lack of data validation from other institutions; (2) the sample size and variables of the training set used to construct the model still need to be increased; (3) the data used to train the model in this study came from only one institution; and (4) the predictive results of the model still need to be prospectively validated in future clinical practice.

## Conclusion

Clinical screening and management of independent risk factors for bowel resection in patients with incarcerated inguinal hernia should be strengthened, and preventive measures should be actively implemented. The predictive model developed in this study has high diagnostic efficacy for bowel resection associated with incarcerated inguinal hernia, with the aim of reducing the incidence of bowel resection and unplanned secondary surgery.

## Data Availability

The datasets generated during the current study are not publicly available due to the limitations of hospital regulations but are available from the corresponding author on reasonable request.

## Abbreviations

IGH: Incarcerated groin hernia
DCA: Decision curve analysis
C-index: Consistency index
COPD: Chronic obstructive pulmonary disease
NRL: Neutrophil to lymphocyte ratio
ROC: Receiver Operating Characteristic Curve
AUROC: Area Under the ROC Curve
